# Salvage of thrombosed arteriovenous fistulae of patients on hemodialysis: report on the experience of a Brazilian center

**DOI:** 10.1590/2175-8239-JBN-2018-0036

**Published:** 2018-08-30

**Authors:** Ricardo Portiolli Franco, Domingos Candiota Chula, Marcia Tokunaga de Alcantara, Eduardo Camargo Rebolho, André Ricardo Ampessan Melani, Miguel Carlos Riella

**Affiliations:** 1 Fundação Pró-Renal, Centro de Nefrologia Intervencionista, Curitiba, PR, Brasil.

**Keywords:** Dialysis, Arteriovenous Fistula, Radiology, Interventional, Angioplasty, Thrombolytic Therapy, Venous Thrombosis, Endovascular Procedures, Diálise, Fístula Arteriovenosa, Radiologia Intervencionista, Angioplastia, Terapia Trombolítica, Trombose Venosa, Procedimentos Endovasculares

## Abstract

**Introduction:**

Hemodialysis vascular access thrombosis is an acute event that can interrupt the dialytic treatment. A timely management can restore access patency, avoiding the use of central venous catheters and their complications.

**Objective:**

To present the experience from a Brazilian Interventional Nephrology Center (INC) in the salvage of arteriovenous fistula (AVF) and grafts for hemodialysis.

**Methods:**

A retrospective study was performed to evaluate the primary and secondary patencies of 41 hemodialysis accesses with thrombosis confirmed by ultrasound and submitted to endovascular salvage procedures. We considered clinical success the use of the access for at least 3 subsequent hemodialysis sessions. The procedures were done in an outpatient center by interventional nephrologists. Patients were followed for up to 18 months with Doppler every 3 months.

**Results:**

Forty-five salvage procedures were performed in 41 accesses of 40 hemodialysis patients with native AVF or grafts. Of these, 90% were AVF, mostly upper arm, and 10% were grafts. Clinical success rate was 60% (27 procedures). Primary patency at 12 months was 39% and secondary was 52%. Gender of the patient, diabetes, and location of the access did not correlate statistically with outcomes. There were 3 major complications (anastomosis rupture, grade 3 hematoma, and anaphylactic shock).

**Conclusion:**

The majority of thrombosed accesses can be successfully treated, maintaining its long-term patency. The need of repeated intervention is frequent.

## RESUMO

**Introdução:**

A trombose dos acessos vasculares para hemodiálise é um evento agudo que interrompe o tratamento dialítico. O manejo em tempo hábil pode restaurar a patência do acesso, evitando o uso de cateteres centrais e suas complicações.

**Objetivo:**

Apresentar a experiência brasileira de um centro de nefrologia intervencionista no salvamento de fístulas arteriovenosas (FAV) e próteses para hemodiálise.

**Métodos:**

Estudo retrospectivo, avaliando as patências primária e secundária de 41 acessos para hemodiálise com trombose confirmada por ultrassonografia e submetidos a salvamento por via endovascular. Consideramos sucesso clínico o uso do acesso por no mínimo 3 sessões de hemodiálise. Os procedimentos foram realizados em regime ambulatorial por nefrologistas intervencionistas. Os pacientes foram acompanhados por até 18 meses com Doppler trimestral.

**Resultados:**

Foram realizados 45 procedimentos de salvamento em 41 acessos de 40 pacientes em hemodiálise por FAV ou prótese. 90% dos acessos abordados foram FAV, sendo a maioria proximais, e 10%, próteses. A taxa de sucesso clínico foi de 60% (27 procedimentos). A patência primária em 12 meses foi de 39% e a secundária, de 52%. O gênero, presença de diabetes e localização do acesso não se correlacionaram significativamente com os desfechos avaliados. Ocorreram 3 complicações maiores (rotura de anastomose, hematoma grau III e choque anafilático).

**Conclusões:**

A maioria dos acessos com trombose pode ser tratada, mantendo sua patência em longo prazo. É frequente a necessidade de intervenções repetidas.

## INTRODUCTION

Arteriovenous fistula (AVF) is considered the access of choice for hemodialysis due to its lower rates of infectious and noninfectious complications and lower costs of treatment compared to grafts and central catheters.^1^ The thrombosis of a definitive access for hemodialysis, be it a fistula or graft, is an acute event that can interrupt dialysis treatment, with need for catheter insertion if untreated. The major cause of AVF thrombosis is the presence of venous stenosis due to intimal hyperplasia, causing low flow and finally access thrombosis.^2^^,^ 3 The treatment of AVF thrombosis can be surgical with thrombectomy or pharmacological with thrombolytics, followed by angioplasty of the causative stenosis, with similar results between these modalities.^4^^-^ 9 In Brazil, there are few centers with immediate access to fluoroscopy and trained personnel for the treatment of AVF thrombosis, and current management is usually the implantation of a catheter until the creation of a new access. This practice, in addition to depleting the patient's vascular bed, seems to be costlier to the health care system due to a greater need for intervention and hospitalization in patients with catheters.^10^ We describe here the initial experience of the *Interventional Nephrology Center (INC) of Pró-Renal Brazil Foundation* in the management of thrombosis of vascular access through pharmacological thrombolysis followed by angioplasty.

## MATERIALS AND METHODS

### STUDY DESIGN

This was a retrospective study using prospectively tabulated data from the vascular access program of the INC of Pró-Renal Brazil Foundation.

### PATIENT POPULATION

This INC assists approximately 700 patients of four chronic hemodialysis clinics, and as of March 2014, the center has implemented a vascular access program, consisting of Doppler access surveillance, venous stenosis angioplasty, and pharmacological thrombolysis followed by angioplasty of thrombosed access.

We included 40 patients on hemodialysis with vascular access thrombosis who underwent pharmacological thrombolysis from August 2014 to January 2017.

### PROCEDURES

Salvage procedures were performed at INC in an outpatient setting, with patients being discharged on the same day. Access was evaluated by ultrasonography in the immediate preoperative period to confirm the diagnosis of thrombosis, and evaluate the thrombus extension and presence of venous stenosis. The contraindications to salvage were history of resistant or intractable stenosis in previous angioplasty and large thrombus volume (for example, an aneurysmatic cephalic vein with thrombus filling its whole extension in the arm).

**Figure 1:**
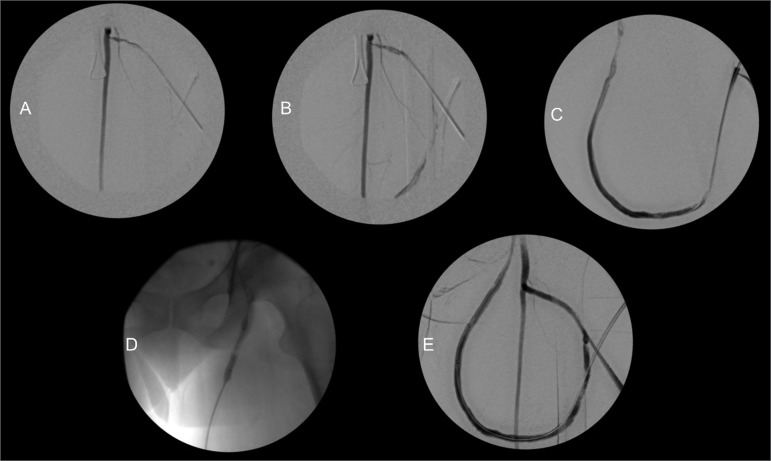
Femoral graft thrombolysis guided by angiography. A, Contrast injection through catheter in the arterial anastomosis showing flow only in the femoral artery, without contrasting the graft. B and C, After alteplase infusion in the arterial anastomosis there is return of partial flow in the graft. D, Angioplasty of the venous anastomosis. E, Final result showing return of flow in the graft.

**Figure 2:**
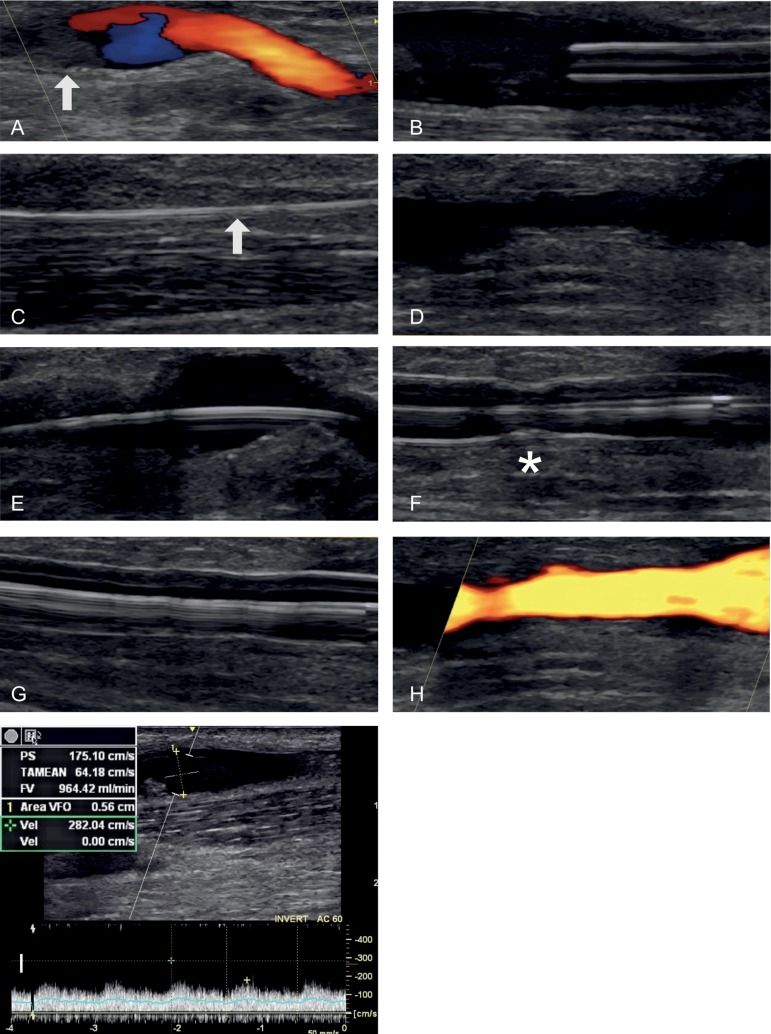
AVF thrombolysis and angioplasty guided by ultrasound (US). A, Juxta-anastomotic region US showing flow on color Doppler and echogenic thrombus (arrow) in the AVF. B, US showing vascular introducer inside the AVF. C, Hydrophilic guidewire passing through the thrombus (arrow). D, After alteplase infusion the thrombus was dissolved. US showing absence of thrombus on the stenotic region. E, F, G, Sequential images of balloon angioplasty. On picture F, the presence of a "waist" on the balloon (*) confirms the presence of stenosis. H, Confirmation of blood flow on the AVF on power Doppler. I, Intra-access blood flow quantified in 964 mL/min on spectral Doppler after procedure.

The procedure consisted briefly of puncture of the AVF, insertion of a 7F introducer by Seldinger technique and passage of a hydrophilic guide through the stenosis. Next, infusion of 1 mg/mL alteplase (Actilyse^®^, Boehringer Ingelhein, Germany) via the catheter or by the introducer was done in the region near the anastomosis. Serial angiography and transoperative ultrasonography were performed to monitor thrombus resolution and the stenosis (Figure 1). After return of flow or partial resolution of the thrombus, angioplasty of the culprit lesion with a non-compliant balloon (Mustang^®^, Boston Scientific, USA) was performed. As an exception, two salvage procedures were guided exclusively by ultrasonography (Figure 2) and not by fluoroscopy.

In cases of successful salvage but with residual cloth or a thrombus resistant to angioplasty, patients were maintained on acetylsalicylic acid 100 mg and clopidogrel bisulfate 75 mg until resolution of the thrombi, verified by ultrasonography during follow-up.

The use of hemodialysis access for at least 3 subsequent sessions was considered a clinical success.

### FOLLOW-UP

Patients were reassessed within 15 days after thrombolysis and placed on quarterly Doppler access surveillance for intra-access blood flow measurement and diagnosis of stenosis. New angiography was indicated in cases of a blood flow < 600 mL/min or a 25% drop in relation to the baseline, or clinical alteration, such as difficulty in puncture or prolonged bleeding, associated with a diagnosis of stenosis > 50% on Doppler.

### OUTCOMES

Access survival was evaluated by Kaplan Meier curves. Primary patency and secondary patency were defined based on the most recent guidelines.^11^

### STATISTICAL ANALYSIS

The chi-square test with a significance level of 0.05 was used to evaluate the correlation between patient characteristics (diabetes, gender, access site, or age group) with the success or failure of the procedure. Kaplan Meier non-parametric estimator was used for the survival functions, where the differences between the survival curves were considered significant if *p* < 0.05, using the Log Rank test. Patients who died, underwent transplantation, switched between dialysis method or centers with functioning access were censored from the analysis.

## RESULTS

In the period from August 2014 to January 2017, 41 accesses with confirmed diagnosis of thrombosis from 40 patients undergoing hemodialysis were submitted to a total of 45 procedures.

Male patients comprised 62.5% of the sample; mean age was 57.4 ± 18.0 years. As for comorbidities, 75% of the patients had hypertension, 40% diabetes, 10% coronary artery disease, and 7.5% history of stroke. The majority of accesses were AVF (90%), 56% were proximal fistulas (brachiocephalic, brachio-median, and brachiobasilic), 34% were distal fistulas (wrist radiocephalic) and 10% were grafts. Thirteen accesses (28%) were less than 3 months old, and 84% were in use for up to 24 months. The earliest approached access had 3 weeks since creation and the oldest 51 months.

All patients presented thrombosis of the vascular access confirmed by ultrasonography and were submitted to pharmacological thrombolysis followed by angioplasty. Considering the number of procedures performed, 27 presented a successful thrombolysis outcome, 17 unsuccessful, and one presented AVF occlusion, precluding the progression of the material and treatment. For outcomes calculation, the case with occlusion was considered an unsuccessful procedure, totaling 18 unsuccessful and 27 successful procedures, with a success rate of 60%. Patients were followed for up to 18 months. The presence of diabetes, patient's gender, the site of the fistula, and the age group did not correlate significantly with the procedure success.

### SURVIVAL ANALYSIS

**Figure 3:**
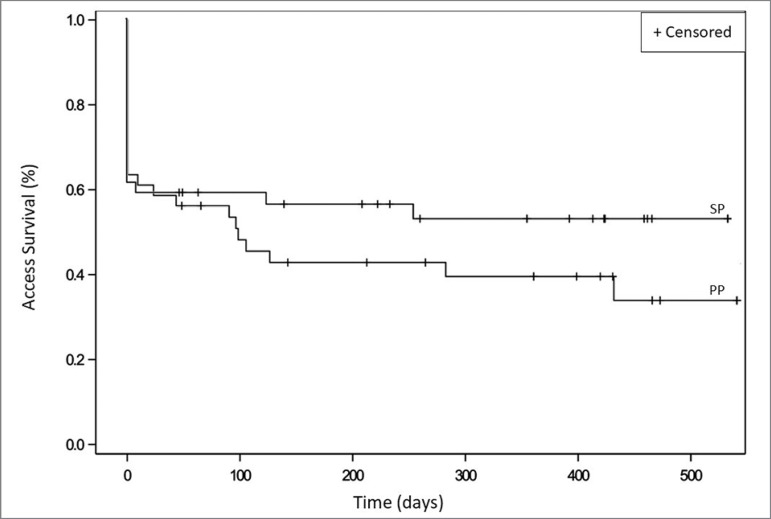
Kaplan Meier vascular access survival curves according to the primary patency (PP) and secondary patency (SP).

Patients with successful and unsuccessful thrombolysis at baseline were included for analysis of the survival of the accesses. Figure 3 shows the survival curves for the primary and secondary patency of the accesses.

## PRIMARY PATENCY ANALYSIS

Of the 41 fistulas, 25 presented primary patency outcome (new angioplasty, death or loss of access) in the period. The primary patency of the accesses, that is, no need for new intervention or loss of access at 3, 6, 12, and 18 months was 53, 42, 39, and 33%, respectively.

### SECONDARY PATENCY ANALYSIS

During the 18-month follow-up, 15 accesses were abandoned due to failure of the initial procedure and 4 due to definitive access thrombosis after initial thrombolysis. Secondary patency, that is, functional accesses in use, independent of new angioplasty or thrombolysis at 3, 6, 12, and 18 months was 58, 55, 52, and 52%, respectively.

If we consider only the accesses submitted to successful initial thrombolysis, that is, 60% of the cases, the secondary patency at 18 months was 80.63%.

## COMPLICATIONS

A total of 9 complications (20%) occurred, of which 3 (6%) were major complications, being one grade III hematoma, one anastomosis rupture, and a anaphylactic shock; the last two with need for hospitalization. There were 4 cases of grade I hematomas, that is, without access impairment. One patient presented grade III hematoma, due to perforation of the cephalic arch, which was occluded. This case was managed with compression and suspension of the procedure, without need of hospitalization, but with definitive loss of access and need for a catheter. There was a case of symptomatic hand arterial embolism, with pain during the procedure, treated with infusion of alteplase in the radial artery and improvement of the symptoms, without need for hospitalization or functional impairment. Two patients had significant clinical complications, one with anaphylactic shock, probably due to radiocontrast infusion, requiring intubation and ICU admission and another with bacteremia after the start of the procedure probably due to infected thrombus in the AVF, necessitating the suspension of thrombolysis. This patient did not need hospitalization. A case of anastomosis rupture occurred during early approach of a thrombus in a juxta-anastomotic region. This patient was approached at 3 weeks after AVF creation and we choose to perform the procedure due to the absence of adequate veins to make a new AVF. In this case, surgery was needed, which was performed by the team's vascular surgeon at the outpatient surgical center, the patient was kept hospitalized for observation. There was no functional impairment.

There were 3 deaths during the follow-up, however none was related to the procedures and all were after more than 30 days of the intervention.

## DISCUSSION

The experience reported here in hemodialysis access salvage, demonstrates that these cases can be managed by the nephrologist on an outpatient basis, provided that the necessary structure and trained staff are in place. The outpatient treatment strategy for access dysfunction is significantly cheaper than the inpatient strategy, leading to less missed dialysis sessions and less need for hospitalization.^12^^-^ 13

In our study, in which most accesses were native AVF (90%), we used the endovascular technique of pharmacologic thrombolysis and subsequent angioplasty of the venous stenosis. The literature shows a success rate of 76 to 94%,^5^^-^ 7 ^,^ 14 ^-^ 18 with a greater chance of success for distal fistulas in some studies.^7^^,^ 14

Table 1 provides data from the most recent literature. In our study, there was no difference in success between the different types of fistulae. However, we achieved a clinical success rate of 60%, below the previously mentioned studies. This may be due to the learning curve and to the fact that clinical success, that is, feasibility of hemodialysis, was considered the outcome, in contrast to the radiological success in some studies. However, despite the apparent lower initial success, our patency rates are close to other similar studies. In the two largest series of cases published with native arteriovenous fistulae performed at different centers by Nassar and Nikam, primary patency at 12 months was achieved in 17 and 44% of cases, respectively.^14^^,^ 15 Our primary patency at 12 months was 39%, showing that the majority of accesses required reintervention. In the Nikam study, which used a technique very similar to ours, there is an important bias for comparison, since it included cases of acute failure due to severe stenosis generating very low flow and preventing access use, but without thrombus (42%), and cases with confirmed thrombosis (58%). In this important study, the presence of thrombosis conferred a worse primary and secondary patency to cases submitted to salvage (HR 1.9 / 95%CI, 1.2-3), which may explain our inferior results, since all cases presented here had thrombosis confirmed by ultrasonography. Regarding secondary patency, our results at 3, 6, and 12 months were 58, 55, and 52%, respectively. The literature shows secondary patency rates of 44 to 89% at 12 months.^5^^,^ 15 ^,^ 16 ^,^ 19 The interpretation of these data is that, in our series, more than half of the accesses with thrombosis and submitted to salvage attempts continued to be used for up to 12 months, which reduces the need for central catheters.

**Table 1:** Summary of recent bibliographic references.

Author (year)		Specialty		Prospective study		N		AVF		Graft		Objective		Thrombolytic use?		Time since trombose		Device		Technique(s)		Success		Primary Patency		Secondary Patency		Complications		Conclusions		Notes
Majority Graft																																
Nassar (2015)		Nephrologist		404 retrospective cases 116 prospective		520 procedures 465 patients		100%		0%		To report a cohort results of 520 cases of AVF thrombosis in 8 years.		Ocasional *Doesn't cite %		-		-		Mechanical Thrombectomy (Embolectomy / Thromboaspiration / Angioplasty)		91%		1 m: 80% 3 m: 60% 6 m: 40% 12 m:, 17% 24 mo: 0,06%		**** Assisted Primary Patency 1 m: 87% 3 m: 81% 6 m: 73% 12 m:, 54% 24 mo: 37%		Total 1.3% 0.8% major 7 ruptures 3 AVF losses due to grade III hematoma.		Salvage is possible in 90% of cases, with low rate of complications. Distal AVF had greater patency than proximal ones."		Outpatient procedures
Nikam (2015)		Radiologist		Yes		410 procedures 232 patients		73%		27%		Long-term results of salvage of AVF / grafts with acute dysfunction. * 58% with confirmed thrombosis.		16%		-		AngioJet / Tretola (15%)		Maceration / Angioplasty 59% Mechanic (Angiojet, Tretola)15% Pharmacomechanic (RTPA) 16%		94% (AVF) 92% (Graft)		AVF: 1 m: 82% 6 m: 64% 12 m: 44% 24 m: 34% 36 m: 26% Graft: 1 m: 50% 6 m,: 14% 12 m: 8%		-		Total 6% 2 grade II hematomas 1 Bacteremia Arterial embolism 1,2% No pulmonary embolism		Balloon maceration (preferred technique in the study) is safe and cost-effective.		Aspirin associated with greater primary and secondary patency. Presence of thrombosis was a risk factor for loss of primary and secondary potencies.
Moossavi (2007)		Radiologis		Yes		49 patients 49 accesses		100%		0%		To determine the success of endovascular salvage of arteriovenous fistula thrombosis.		Yes		< 48hs		AngioJet		Mechanical Thrombectomy (Endovascular / Angioplasty)		96%		1 m: 85% 6 m: 55% 12 m:, 50% 24 mo: 43%		1 m: 97% 6 m: 95% 12 m:, 75% 24 mo: 55%		8,4% 2 grade I hematoma 2 partial embolisms		In cases of fistula thrombosis, 96% can have their patency restored if the salvage is performed in 48 hours.		Inpatient procedures
Turmel- Rodrigues (2000)		Radiologist		Yes		93 AVF procedures 168 graft procedures 151 accesses		48%		52%		To study the safety and effectiveness of percutaneous salvage of thrombosed hemodialysis accesses.		Ocasional, Urokinase * "Lyse and wait”, doesn’t cite %		< 72hs *Except 2 cases		-		Mechanical Thrombectomy (Thromboaspiration / Angioplasty)		93% proximal AVF 76% distal AVF *Clinical Succes		AVF proximal: 12 m: 49% AVF distal 12 m: 9% Grafts 12 mo: 14%		AVF proximal: 12 m: 81% AVF distal 12 m: 50% Grafts 12 mo: 83%		1 pulmonary embolism 1 acute Pseudoaneurism 1 major bleeding		Percutaneous salvage by manual catheter thromboaspiration is effective in more than 90% of cases, with better results for distal fistulas."		Outpatient procedures
Lee (2014)		Nephrologist		No		75 procedures 42 patients		11%		89%		To evaluate the results of percutaneous thrombectomy performed by Nephrologists.		Yes		< 24 hs		-		Pharmacomechanic Thrombectomy (Endovascular / Angioplasty		89%		1 m: 79% 3 m: 56% 6 m:, 25% *Included only cases with primary success.		1 m: 92% 3 m: 85% 6 m:, 83% *Included only cases with primary success.		Total 6,6% 0% major 2 hematomas 2 arterial embolisms		Percutaneous thrombectomy by interventional Nephrologist is safe and effective.		Inpatient procedures
Ponce (2014)		Surgeon and Nephrologist		Yes		354 procedures 336 acessos		0%		100%		To compare success rate of surgical and endovascular salvage in grafts.		Yes		< 24 hs		Arrow Tretola		Endovascular Salvage (n=126) / Surgery (n=228)		87% / 100%		1 m: 74% / 74% 3 m : 63% / 67% 6 m: 53% / 55%		-		Doesn’t cite		The result of both techniques is comparable.		Outpatient procedures

In our study, the vast majority of cases were treated within 48 hours of thrombosis, with the later cases being treated after 7 and 30 days. A therapeutic window considered good is an interval of 24 to 48 hours after thrombosis, although there is divergence in the literature, with some authors showing good results even after this period.^20^^,^ 21

Access salvage techniques include surgical thrombectomy, endovascular thrombectomy associated with angioplasty of stenosis with or without thrombolytics, and the use of specific devices for thrombus aspiration and lysis of clots (Angiojet^®^, Boston Scientific, USA/Arrow-Terotola^®^, Arrow Medical, England/Aspirex^®^, Straub Medical, Switzerland). It is agreed that almost all thrombosis are related to venous stenosis and that these should be treated in order to avoid early recurrence.^1^ In the most recently published case series, only 1% of thrombosis were not associated with any angiographic findings and only 3% were not related to stenosis.^14^^,^ 15 Therefore, even if a surgical thrombectomy is performed, the correction of venous stenosis is essential. In a meta-analysis published in 2009, the 1-year secondary patency for endovascular salvage, as performed by us, ranged from 50 to 89%.^6^ This study compared the results of surgical and endovascular techniques and concluded that the two modalities are comparable in the treatment of graft thrombosis, with an advantage for long-term surgical treatment only in native wrist fistulas.

Of note, Turmel-Rodrigues^7^ found a better secondary patency at 12 months in the distal versus the proximal fistulae (81 *vs*. 50%). In our study, although we found a difference in secondary patency with advantage for distal fistulae, it did not reach statistical significance (*p* = 0.3012).

The importance of rescuing accesses for hemodialysis was very well explained by Coentrao,^10^ who compared a historical cohort in which patients with vascular access thrombosis were submitted to catheter implantation and new AVF creation to a group of patients undergoing percutaneous thrombectomy followed by angioplasty. After 6 months of follow-up, 91% of the patients in the thrombectomy group were receiving hemodialysis through an AVF, compared to only 33% of the historic cohort of catheters, although most (22 of 24 patients) underwent a new AVF. This difference may also be due to the effect of saving accesses and to other factors involved after the implementation of a better care with the vascular accesses in relation to the historical cohort. We observed a similar finding in our service after the introduction of elective angioplasty of accesses with dysfunction and Doppler surveillance, which reduced the need for new fistulae. Furthermore, in this analysis, the author compared the costs of the thrombectomy versus the catheter approach, concluding that the costs where significantly higher for the second group (mean in USD 375 *versus* 706, *p* = 0.048), with the largest expenses due to hospitalizations. In the thrombectomy group, the main expense was the procedure itself (USD 232).

In our series, minor complications occurred in 13% (6 cases) of the procedures and major complications in 6% (3 cases). Two patients with major complications required hospitalization (4%), one due to anaphylactic shock and another due to rupture of the arteriovenous anastomosis requiring surgery. Both were discharged within a few days. We have considered our complication rate as high, especially the major ones. Complication rates are higher in cases of access thrombolysis in relation to elective angioplasty. The literature shows a complication rate of up to 8.4% depending on the technique used. 7 ^,^ 14 ^,^ 15 ^,^ 16 ^,^ 22. Several studies cited the performance of the procedures by interventional nephrologists 8 ^,^ 14 ^,^ 18 ^,^ 22 ^,^ 23 ^,^ 25, with complication rates similar to other specialists.

Up to the present time and to the best of our knowledge, this is the first series of cases of vascular access salvage performed by nephrologists in Brazil. The procedures were performed on an outpatient basis, opening the possibility of a more agile and less costly care system. One of the strengths of our study is that all thrombosis were confirmed by ultrasonography excluding cases with only severe stenosis and low flow, which may possibly have a better outcome and better prognosis.

As negative points, this is a retrospective study, with a small number of patients, which evaluated the first salvage procedures performed by our interventional nephrologists, and results reflect the learning curve of the team.

We conclude that vascular access salvage procedures can be performed by nephrologists on an outpatient basis after specific training, reaching complication rates and long-term access patency consistent with the existing literature. Salvaged accesses reduce the need for central catheters, and possibly morbidity and associated costs. However, there is a frequent need for repeated procedures to maintain these accesses.
